# A graphical vector autoregressive modelling approach to the analysis of electronic diary data

**DOI:** 10.1186/1471-2288-10-28

**Published:** 2010-04-01

**Authors:** Beate Wild, Michael Eichler, Hans-Christoph Friederich, Mechthild Hartmann, Stephan Zipfel, Wolfgang Herzog

**Affiliations:** 1 Department of General Internal Medicine and Psychosomatics, Medical University Hospital Heidelberg, Heidelberg, Germany; 2 Department of Quantitative Economics, Maastricht University, Maastricht, The Netherlands; 3 Department of Psychosomatic Medicine and Psychotherapy, Medical University Hospital Tübingen, Tübingen, Germany

## Abstract

**Background:**

In recent years, electronic diaries are increasingly used in medical research and practice to investigate patients' processes and fluctuations in symptoms over time. To model dynamic dependence structures and feedback mechanisms between symptom-relevant variables, a multivariate time series method has to be applied.

**Methods:**

We propose to analyse the temporal interrelationships among the variables by a structural modelling approach based on graphical vector autoregressive (VAR) models. We give a comprehensive description of the underlying concepts and explain how the dependence structure can be recovered from electronic diary data by a search over suitable constrained (graphical) VAR models.

**Results:**

The graphical VAR approach is applied to the electronic diary data of 35 obese patients with and without binge eating disorder (BED). The dynamic relationships for the two subgroups between eating behaviour, depression, anxiety and eating control are visualized in two path diagrams. Results show that the two subgroups of obese patients with and without BED are distinguishable by the temporal patterns which influence their respective eating behaviours.

**Conclusion:**

The use of the graphical VAR approach for the analysis of electronic diary data leads to a deeper insight into patient's dynamics and dependence structures. An increasing use of this modelling approach could lead to a better understanding of complex psychological and physiological mechanisms in different areas of medical care and research.

## Background

In recent years, the use of electronic diaries in clinical research has become increasingly popular [1,2]. In many different medical areas--such as neurology [3], sleep medicine [4], paediatrics [5], dermatology [6], gynaecology [7], psychosomatic medicine [8], and rheumatology [9]--electronic diary data provide new insights into processes and temporal relationships. Particularly, in clinical practice and research regarding chronic pain electronic diary assessment is frequently used to examine day-to-day variation in symptoms as well as to investigate the impact of constant self-monitoring on the patient's behaviour [10-12]. Also, in psychosomatic research, the application of electronic diaries is of particular interest to mirror development processes over time and to capture complex dynamical systems with several interacting variables [13]. For instance, in patients with fibromyalgia, pain symptoms early in the day show associations with salivary cortisol levels [14]. As in studies with healthy control persons, elevated cortisol levels have been associated with increased negative mood, anxiety, and fear, it could be possible that cortisol influences pain via mood-induced alterations. However, to date, the dynamic dependence structure of these interacting variables still remains unclear.

In general, in psychosomatic research--but also in other medical domains--there is an urgent need to analyse treatment courses in order to optimise treatment and prevent relapse. Recent randomized controlled trials mirror this necessity by including longitudinal process measures in addition to the standard pre-post-outcome measurements [15-17]. Electronic diaries can be used as a powerful and efficient tool for the investigation of processes and associations between physical symptoms and psychosocial variables. Nevertheless, a possible drawback of using electronic diaries with daily or even more frequent monitoring is that high motivation is required from the patients to complete the study. Also, for completers, the daily monitoring may become burdensome, which can cause a bias in the data collection. To ensure continuing patient compliance, periodic feedback should be given. However, the use of electronic diaries also reduces the possibility of distortions due to retrospective recall and therefore excludes a bias which is common in retrospective data collection.

Despite the increased availability of electronic diary data and the thus offered opportunity to learn more about the dynamic interrelationships among the variables of interest, appropriate multivariate statistical methods have been applied to date very rarely to the analysis of such data [18]. Most studies use a mixed modelling approach for the analysis of diary data [1]. The application of this approach is limited by the fact that estimation of mixed models is computationally infeasible for multivariate responses of dimension four and larger [19]. Therefore, to be able to answer intriguing questions about dependence structures between several variables and group characteristic processes, more advanced multivariate time series methods are required.

In the past years, Eichler [20,21] presented a new approach for analysing systems of time series that merges the concepts of Granger causality [22] and graphical modelling [23,24]. It is based on vector autoregressive (VAR) models that have been known in multivariate time series analysis for many years [25]. In the research area of functional brain imaging, VAR models have been used in the context of Granger causality for the investigation of directed influences between activated brain areas [26,27]. The new graphical VAR approach extends this method by investigating all possible constrained VAR models, selecting the best fitting model and reflecting it in a clearly directed graph. Therefore, this method offers outstanding possibilities not only to answer important research questions regarding mutual dependencies and temporal group patterns but also to mirror the results in an easily understandable way.

The aim of this article is, first, to give a comprehensive description of the graphical modelling approach for VAR models and, second, to illustrate the relevancy and usefulness of the statistical method by giving a first application to electronic diary data in the psychosomatic research field of binge eating disorder (BED). Binge eating disorder (BED) is characterized by the consumption of large amounts of food over short periods of time, accompanied by a sense of loss of control, and not compensated by inappropriate weight control behaviours [28]. 30-65% of the patients with BED have comorbid obesity involved with an increased risk for subsequent chronic diseases and mortality [29]. The validity of the diagnosis of BED has been contentiously discussed since 1994 [30]. Nevertheless, the association of BED with an extensive psychopathology, impaired quality of life, and social functioning suggests that differentiating BED from obesity and other forms of psychopathology is a critical clinical issue [31,32]. To date, some diary studies exist which investigated the differences between BED and non-BED participants regarding the association between mood and eating behaviour. For instance, Le Grange *et al*. [33] showed that for BED as well as non-BED participants, negative affect and restraint were immediate antecedents of binge eating. In contrast, Greeno *et al*. [34] found different patterns of immediate binge antecedents between patients with and without BED which was replicated by Hilbert and Tuschen-Caffier [35]. However, none of the studies analysed the time series of the patients using a multivariate time series approach. Therefore, no statement could be made regarding feedback loops and dynamic dependence structures of a system of variables. In the present study, we used the graphical vector-autoregressive approach to analyse electronic diary data of 35 obese patients, both with and without a binge eating disorder (BED), who participated in a multi-modal outpatient intervention program [36]. Throughout the course of the treatment period, participants recorded on a daily basis their eating behaviour, levels of depression, anxiety and self-perceived eating control. The aim of the analysis was to investigate the dependence structure between eating behaviour and associated variables for obese patients with and without BED and to find possible differences between these two groups.

## Methods and Results

Before we apply the graphical modelling approach to electronic diary data, we briefly introduce the underlying concepts from multivariate time series.

### Methodological issues

#### Granger causality

Granger-causality [22] is a fundamental tool for the investigation of dynamic interrelationships in multivariate time series. It is based on the common sense conception that causes proceed their effects in time. This temporal ordering implies that the past and present values of a series *X *that influences another series *Y *should help to predict future values of this latter series *Y*. Moreover, this improvement in the prediction of future values should persist after any other relevant information for the prediction has been exploited. This leads to the following definition of Granger-causality: For two time series *X *and *Y *let *Z *be the (vector) time series that comprises all variables that might affect the dependence between *X *and *Y*. We say that *X *Granger-causes *Y *if the current value of *Y *can be better predicted from the past values of all three series *X*, *Y*, and *Z *than from the past values of the two processes *Y *and *Z *alone. Here, "better predicted" means a smaller mean square prediction error. We note that the definition depends on the set of variables *Z *included in the analysis.

In practice, the concept of Granger-causality mostly has been used in the framework of vector autoregressive models for the investigation of linear relationships among the variables.

#### Vector autoregressive models

The vector autoregressive (VAR) model is a straightforward extension of the univariate autoregressive model [25] and describes how the values of the variables at time *t *depend linearly on the values at previous time points. For the sake of simplicity, we restrict ourselves in the following to the simplest case where only the past values at time *t *- 1 are taken into account. Thus a VAR model of order 1--also abbreviated as VAR(1) model--is a linear regression model in which the vector of values at time *t *is regressed on the vector of values at the previous point in time *t *- 1. The model can be thought as a linear prediction model that predicts the current value of a variable based on its own past value on the previous point in time and the past values of the other variables.

When analysing electronic diary data, we are concerned with several vector time series, one for each subject included in the study. For the investigation of the dynamics common to all subjects, we model these vector time series jointly by one common VAR(1) process assuming that there are no dependences between subjects. Thus, if the time series of *K *subjects in *n *variables are given and *X*_*i,k*_(*t*) represents the score of the *k*th person in the *i*th variable at time *t*, we consider the joint regression model

for *i *= 1,..., *n *and *k *= 1,..., *K*, which yields *n*^2 ^regression coefficient *β*_*ij*_, *i*, *j *= 1,..., *n*. For the errors *ε*_*i,k*_(*t*), we assume that they have mean zero and are uncorrelated between different points in time or different subjects, that is,

for all *i *= 1,..., *n*, *k *= 1,..., *K *and

whenever *t *≠ *s *or *k *≠ *l*.

Intuitively, the regression coefficients measure the direct influences of the explanatory lagged variables on the dependent variables. Thus, in the above VAR(1) model, variable *X*_*j *_Granger-causes *X*_*i *_if the coeffcient *β*_*ij *_differs from zero. For the general case of a VAR model of order *p*, we refer to Eichler [20,21,37]. Furthermore, the strength of the direct same-time relationships among the variables is quantified by the entries in the inverse of the variance-covariance matrix--the so-called concentration matrix--of the residuals *ε*_1, *k*_(*t*),..., *ε*_*n*, *k*_(*t*).

The VAR analysis is carried out under the assumption of normality of the data. The method, however, is known to be reasonably robust against departures from the distributional assumptions. In such cases, the fitted model describes the linear relationships found in the data. Furthermore, the assumption of stationarity can be relaxed by defining VAR models with a deterministic or a stochastic trend. Fitting of a deterministic trend basically results in removing the fitted trend whereas a stochastic trend (random walk behavior) does not require special treatment when fitting by least squares or conditional maximum likelihood. These estimation methods do not necessarily require stationarity, which means that time series have time invariant expected values, variances and covariances, but only stationary dynamics in the sense that the internal dependence of the process does not change between different time points. We note that removing of trends frequently is achieved by differencing the series. We do not recommend this practice since in the case of a deterministic trend this will create a serial dependence not previously in the data that cannot be modelled by a VAR process [25,38]. Likewise in the case of a stochastic trend, fitting of a VAR model upon differencing is inadequate if the series are cointegrated [25].

#### Structural modelling approach

The VAR(1) model above allows all lagged and same-time relationships among all variables to be present. By setting certain regression coefficients and entries in the concentration matrix to zero, we obtain a structural VAR model that is associated with a specific pattern of (temporal) interrelationships among the variables. Such constrained VAR models are also called graphical VAR models [21] since their temporal relationships can be visualized by a path diagram or graph. Here, a graph or path diagram consists of a finite set of nodes which are connected by edges depicted as arrows or lines. Each variable of a multivariate time series is represented by a single node [21]. For each regression coefficient that is not constrained to zero a directed edge (i.e. an arrow) pointing from the explanatory variable to the dependent variable is drawn. Undirected edges (i.e. lines) are used to visualize direct contemporaneous relationships among the variables.

The aim of the structural or graphical modelling approach is to determine the constrained VAR model that best describes the pattern of interrelationships present in the electronic dairy data. This best fitting model is identified by an exhaustive search over the space of all possible graphical VAR(1) models. For each model, the parameters are estimated by the conditional likelihood method [25] through an iterative algorithm described in Eichler [37], and the Akaike information criterion (AIC) scores are calculated. Low AIC values reflect model parsimony, favouring a high log likelihood along with a low number of parameters. Measuring the goodness-of-fit of the restricted models by AIC, the model with lowest AIC score is selected as the best fitting model. Instead of the AIC, other model selection criteria such as Bayesian information criterion (BIC) [39] could be used. Furthermore, we note that the standard errors of the parameters are based on the usual asymptotic considerations and as such reasonably robust e.g. against departures from the underlying assumptions. In particular, the asymptotic normality of the quasi-maximum likelihood estimators does not require normally distributed innovations in the VAR model. One problem with the described model selection approach is that, although there may be many models that describe the data almost equally well, it provides only one optimal model. For those dependencies that have been included in the optimal model, this uncertainty can be simply evaluated by significance tests for the corresponding parameters. If the null hypothesis that a particular parameter is zero cannot be rejected, we cannot decide between the optimal model and the smaller model with this parameter constrained to zero. In that case, the corresponding relationship is not well identified by the data and should therefore not be interpreted. This approach, however, does not allow to evaluate the uncertainty of having a parameter falsely constrained to zero. Alternatively, the model uncertainty can be evaluated by comparing all models with lowest AIC score. It has been suggested that models with AIC scores within 2 units of the minimal score should be considered as competitive [40], that is, not statistically significant different from the best model. Finally, we note that in graphical VAR models of an order larger than 1, the significance of edges can be evaluated by Granger causality tests, which simultaneously test for the coefficients at all lags to be zero [25].

#### Estimation of partial contemporaneous and partial directed correlations

After the graphical VAR model with minimal AIC score has been identified, the strength of the links in the model can be assessed by computing the so-called partial directed correlations and partial contemporaneous correlations as measures of strength of the lagged respectively same-time associations between variables [41]. Here, the partial contemporaneous correlation (PCC) is defined as the correlation between two variables at the same point in time after removing the linear effects of the other variables at the same point in time and all variables at previous times. In a VAR(1) model, the partial contemporaneous correlations can be directly computed from the concentration matrix *K*_*ij *_of the residuals.

More precisely, the estimates of the PCCs are given by

Here, the entries  of the concentration matrix are the parameter estimates obtained by fitting the structural VAR model.

As noted above, the direct relationships among the variables across time are modelled in the VAR model by the regression coefficients. Since regression coefficients depend on the unit of measurement, Dahlhaus and Eichler [41] proposed to measure the strength of lagged associations between variables by so-called partial directed correlations (PDC) (see also [20]). In a VAR(1) model, the PDCs measure the linear association between a dependent variable at time t and an explanatory variable at time t-1 after removing the linear effects of all other variables at time t-1. They thus quantify the direct influence of the explanatory variable on the dependent variable. The PDC can be obtained by rescaling the autoregressive coefficients ,

where  are the diagonal entries in the variance-covariance matrix of the residuals. In a graphical VAR model these are estimated by inversion of the concentration matrix . For more details and the case of VAR models of order larger than 1, we refer to Eichler [20].

#### Comparison of graphical VAR models between two subgroups

For the comparison of the dependence structure in the time series data of two subgroups, the data analysis can be performed in three steps. Firstly, in order to determine whether there is a significant overall difference in the temporal relationships between the two subgroups, a common VAR model for all subjects is tested against a VAR model with separate parameters for each group using a likelihood ratio test. Secondly, a structural modelling approach is applied to identify the pattern of temporal relationships for each subgroup. In a last step, the differences in the temporal relationships between two subgroups can be further investigated by comparing the PCCs and PDCs in the respective best fitting reduced models [20].

#### Software

The routines for fitting and selecting constrained VAR models were implemented in the statistical software R http://www.r-project.org.

### Application study in the research field of binge eating disorder

#### Study sample and assessment

We analysed electronic diary data from 35 obese German patients—28 women and 7 men--who took part in a muli-model outpatient intervention program. At the beginning of the treatment, 16 patients fulfilled the DSM-IV diagnostic criteria for BED [28]; the other 19 patients were diagnosed as obese without BED. Over the entire course of the treatment, patients answered questions daily regarding their eating behaviour such as the number of meals consumed each day, the amount of food consumed at each meal, as well as the occurrence of binge eating episodes. The daily assessment of the patients (regarding the number of meals eaten, the amount eaten at each meal and the occurrence of the binge episodes) was used to calculate the variable 'eating behaviour' [42]. The variable 'eating behaviour' is a discrete measurement for the daily amount of food consumed. The correlation between the variable 'eating behaviour' and the occurrence of binge episodes is 0.79. In addition to an assessment of their eating habits, the patients answered questions daily about levels of depression, anxiety, and control over their eating behaviour. The items were rated on a visual analogue scale (VAS) with bipolar anchor statements. The computer program converted the marked points to a numeric scale. Handheld computers of type Palm m100 were used for the electronic diaries. A specific software was developed in Java 2 Micro Edition for the portable computers [43]. An electronic alarm signal daily reminded patients to complete the data assessment.

#### Pre-processing of the diary data

**Figure 1:**
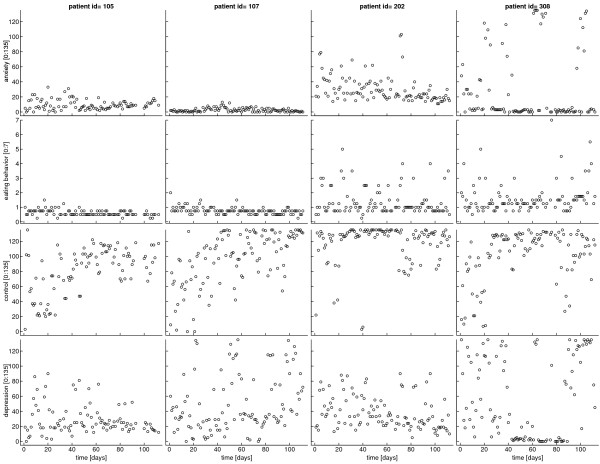
**Raw time series for four patients**. Courses of anxiety, eating behaviour, eating control, and depression during treatment.

Each time series had a length of 112 measurement points, equivalent to 112 days of monitoring. Missing values (6.3%) were replaced using the weighted averages of univariate autoregressive forward and backward predictions. Some of the time series showed a clearly visible trending behaviour, which was removed by subtracting a fifth-order polynomial trend from the individual time series; additionally, each series was standardized by dividing by its standard deviation [44]. In the dynamic analysis of the diary data, we included four variables: eating behaviour (eat), depression (dep), anxiety (anx), and sense of control over eating (ctl). Figure 1 shows exemplary plots of the raw time series of these four variables for four patients. In principle, it would have been interesting to include the treatment variable as an exogenous on-off-variable. However, preliminary to this study the same data were analyzed using a mixed modeling approach to investigate time trends. In these analyses, the treatment variable had been included as a covariate but did not show any significant influence on the eating behaviour or other variables. Therefore, it has been omitted in the present study. In an preliminary analysis, we investigated AR(1) and AR(2) models. Results showed that the structure of the dynamic first-order relationships was not different and the correlations across two days were very weak. As, in addition, a second order model requires estimation of more parameters, we decided for the first-order model.

### Results of structural analysis

Testing a common VAR model for all patients against a VAR model with separate parameters for each subgroup, we found a significant difference in the structure of temporal relationships between the two subgroups (likelihood ratio test: T = 61.04, p = 0.006).

**Figure 2:**
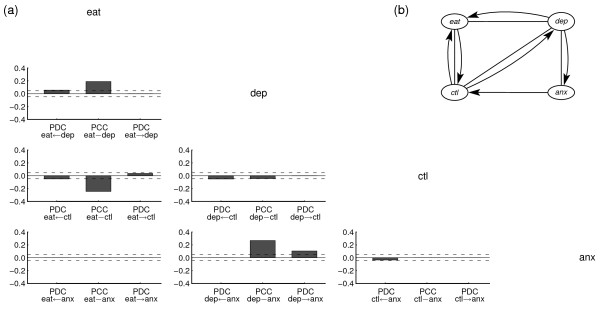
**Structural results for patients with BED**.(a) Partial directed and contemporaneous correlations for best VAR(1) model. Dashed horizontal lines indicate pointwise 95% test bounds for the hypothesis that the PDC respectively PCC is zero. (b) Path diagram associated with best VAR(1) model. Arrows indicate lagged associations; lines indicate contemporaneous associations.

**Figure 3:**
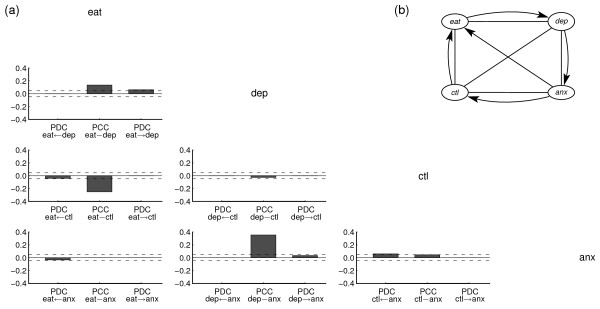
**Structural results for patients without BED**. (a) Partial directed and contemporaneous correlations for best VAR(1) model. Dashed horizontal lines indicate pointwise 95% test bounds for the hypothesis that the PDC respectively PCC is zero. (b) Path diagram associated with best VAR(1) model. Arrows indicate lagged associations; lines indicate contemporaneous associations.

The temporal relationships for the two subgroups identified by the structural modelling approach are visualized by the path diagrams in figures 2 and 3.

The path diagrams reflect the best fitting graphical VAR(1) models for the two subgroups, that is, the models with the lowest AIC score. The partial correlations for the same-day (PCC) and lagged relationships (PDC) in the reduced models are visualized by the bar diagrams in figure 2 and 3. Numeric values of the PCCs and PDCs are shown in table 1 and 2.

**Table 1:** Partial contemporaneous correlations (PCCs) for the optimal VAR(1) models.

Link	eat-dep	eat-ctl	eat-anx	dep-ctl	dep-anx	ctl-anx
Patients with BED	0.190***	-0.244***	--	-0.044	0.268***	--
Patients without BED	0.135***	-0.255***	--	-0.026	0.354***	0.047

Dashed lines instead of a PCC values indicate that in the optimal graphical VAR(1) model this association is constrained to zero.

eat = eating behaviour, dep = depression, ctl = sense of control over eating, anx = anxiety

* *p *< 0.05; ** *p *< 0.01; *** *p *< 0.001

**Table 2:** Partial directed correlations (PDCs) for the optimal graphical VAR(1) models.

		Patients with BED				Patients without BED			
			
To		eat	dep	ctl	anx	eat	dep	ctl	anx
From	eat	0.002	---	0.036	---	0.018	0.063***	---	---
	dep	0.055*	0.182***	---	0.105***	---	0.150***	---	0.034
	ctl	-0.049*	-0.050*	0.266***	---	-0.041	---	0.150***	---
	anx	---	---	-0.035	0.082***	-0.033	---	0.060**	0.103***

Dashed lines instead of a PDC values indicate that in the optimal graphical VAR(1) model this association is constrained to zero.

eat = eating behaviour, dep = depression, ctl = sense of control over eating, anx = anxiety

*p < 0.05; **p < 0.01; ***p < 0.001

For both groups, the tables show that the strongest associations between the variables occurred on the same day. For both groups, the same-day relationship between "eating behaviour" and "depression" is positive (PCC_BED _= 0.19; PCC_Non-BED _= 0.135), which means that high depression scores are related to a high level of eating on the same day. The negative same-day relationship (PCC_BED _= -0.24; PCC_Non-BED _= -0.255) between "eating behaviour" and "eating control" indicates that a low self-assessment of eating control is associated with a high level of eating on the same day. No significant differences were found in the pattern of same-day relationships between the variables for both subgroups. We note that autocorrelations for the variables "depression", "anxiety" and "control over eating" are high and could be depicted by self-loops in the path diagrams. However, for theoretical reasons, these self-loops do not play any role in the graphical analysis of Granger-causal relationships and therefore are usually omitted. Correspondingly, the structural analysis has been carried out without imposing constraints on the autocorrelations.

The most obvious difference between the two subgroups lies in the lagged relationships, that is, the partial directed correlations (PDC) that reflect associations between variables across adjacent days. The positive PDC from depression to eating behaviour in the group with BED (PDC_BED _= 0.055) indicates that, for obese patients with BED, after a day of high depression, a high level of eating becomes more probable. One could interpret the results to mean that for obese patients with BED, eating becomes a dysfunctional mean to regulate emotions and to compensate for a bad previous day. In contrast, obese patients without BED show the opposite dependency; in this group, a high level of eating on any one day predicts a higher depression level on the following day. Additional smaller differences between the two subgroups are shown in Table 2.

For an assessment of the uncertainty about the selected optimal model, Tables 3 and 4 list for each subgroup the 25 models with the lowest AIC score. On the one hand, these lists confirm the conclusions drawn from significance tests on the unconstrained coefficients: For patients with BED, we consistently detect that "eating behaviour" Granger-causes "depression" whereas for patients without BED the relationship is reversed. On the other hand, the tables indicate a large uncertainty about many lagged relationships which indicates that the amount of data is not sufficient for identifying the complete pattern of relationships among the variables.

**Table 3:** List of graphical VAR(1) models with lowest AIC scores for patients with BED.

	eat	eat	eat	dep	dep	dep	ctl	ctl	ctl	anx	anx	anx	eat	eat	eat	dep	dep	ctl	
	↓	↓	↓	↓	↓	↓	↓	↓	↓	↓	↓	↓	|	|	|	|	|	|	
	dep	ctl	anx	eat	ctl	anx	eat	dep	anx	eat	dep	ctl	dep	ctl	anx	ctl	anx	anx	AIC
1		•		•		•	•	•				•	•	•		•	•		0.000
2		•		•		•	•	•	•			•	•	•		•	•		0.128
3		•		•		•	•	•			•	•	•	•		•	•		0.177
4	•	•		•		•	•	•				•	•	•		•	•		0.288
5		•		•		•	•	•	•		•	•	•	•		•	•		0.293
6	•			•		•	•	•				•	•	•		•	•		0.356
7	•	•		•		•	•	•	•			•	•	•		•	•		0.416
8				•		•	•	•				•	•	•		•	•		0.456
9	•			•		•	•	•	•			•	•	•		•	•		0.483
10	•	•		•		•	•	•			•	•	•	•		•	•		0.495
11	•			•		•	•	•			•	•	•	•		•	•		0.576
12				•		•	•	•	•			•	•	•		•	•		0.582
13	•	•		•		•	•	•	•		•	•	•	•		•	•		0.611
14		•		•		•	•	•					•	•		•	•		0.617
15				•		•	•	•			•	•	•	•		•	•		0.641
16	•			•		•	•	•					•	•		•	•		0.669
17	•			•		•	•	•	•		•	•	•	•		•	•		0.689
18		•		•		•	•	•	•				•	•		•	•		0.748
19				•		•	•	•	•		•	•	•	•		•	•		0.755
20				•		•	•	•					•	•		•	•		0.768
21	•			•		•	•	•	•				•	•		•	•		0.798
22	•	•		•		•	•	•					•	•		•	•		0.883
23				•		•	•	•	•				•	•		•	•		0.897
24	•			•		•	•		•			•	•	•		•	•		0.923
25		•	•	•		•	•	•				•	•	•		•	•		0.939

The dots indicate the directed and undirected edges of the path diagrams determining the models. AIC scores are given relative to the lowest score.

**Table 4:** List of graphical VAR(1) models with lowest AIC scores for patients without BED.

	eat	eat	eat	dep	dep	dep	ctl	ctl	ctl	anx	anx	anx	eat	eat	eat	dep	dep	ctl	
	↓	↓	↓	↓	↓	↓	↓	↓	↓	↓	↓	↓	|	|	|	|	|	|	
	dep	ctl	anx	eat	ctl	anx	eat	dep	anx	eat	dep	ctl	dep	ctl	anx	ctl	anx	anx	AIC
1	•					•	•			•		•	•	•		•	•	•	0.000
2	•				•	•	•			•		•	•	•		•	•	•	0.041
3	•			•		•	•			•		•	•	•		•	•	•	0.213
4	•	•				•	•			•		•	•	•		•	•	•	0.346
5	•	•		•		•	•			•		•	•	•		•	•	•	0.554
6	•					•	•			•	•	•	•	•		•	•	•	0.657
7	•						•			•		•	•	•		•	•	•	0.679
8	•				•	•	•			•	•	•	•	•		•	•	•	0.683
9	•					•	•					•	•	•		•	•	•	0.775
10	•				•	•	•					•	•	•		•	•	•	0.817
11	•	•			•	•	•			•		•	•	•		•	•	•	0.825
12	•						•			•	•	•	•	•		•	•	•	0.837
13	•					•	•				•	•	•	•		•	•	•	0.864
14	•				•	•	•				•	•	•	•		•	•	•	0.888
15	•				•	•	•			•		•	•	•		•	•	•	0.933
16	•	•					•			•	•	•	•	•		•	•	•	1.001
17	•			•	•	•	•			•		•	•	•		•	•	•	1.007
18	•			•		•	•			•	•	•	•	•		•	•	•	1.023
19	•	•					•			•		•	•	•		•	•	•	1.041
20	•						•				•	•	•	•		•	•	•	1.044
21	•				•		•			•	•	•	•	•		•	•	•	1.052
22	•			•			•			•		•	•	•		•	•	•	1.085
23	•					•	•					•	•	•		•	•	•	1.103
24	•	•				•	•				•	•	•	•		•	•	•	1.196
25	•	•					•			•	•	•	•	•		•	•	•	1.198

The dots indicate the directed and undirected edges of the path diagrams determining the models. AIC scores are given relative to the lowest score.

## Discussion and conclusion

In medical research, electronic diaries are widely used to measure fluctuations in patients' symptoms and to reflect developments over treatment courses. Using the graphical VAR approach to model electronic diary data has several outstanding advantages. The diary data of whole patient groups are analysed simultaneously and dynamic dependence structures of two different patient groups can be compared. Feedback mechanisms can be modelled and information about influence directions between variables is provided. Furthermore, dynamic dependencies among variables are visualized in a clearly directed graph. Finally, the statistical techniques required for fitting the models can be easily and efficiently implemented, which allows estimating large numbers of models in reasonably short computing time.

We note that the graphical modelling approach presented here is not restricted to VAR models but could also be used for dynamic non-linear time series models very similar to the mixed models commonly used in the analysis of longitudinal data. However, estimation of mixed models for multivariate response of dimension four or larger is computationally not feasible. Other modern approaches such as the one by Fieuws *et al*. [19], which uses pairwise modelling, are not suitable when one is interested in the multivariate dynamic (in the sense of Granger-causality) interrelationships among the variables. Moreover, we are not only interested in the dynamic interrelationships (as represented by the autoregressive coefficients in our model) but also in the contemporaneous associations. For this, it is necessary to impose conditional independence constraints on the distribution of the multivariate response. For mixed models, the authors are not aware of a feasible solution to this problem.

Dahlhaus [45] proposed another interesting approach to the analysis of multivariate time series using graphical interaction models. This method is based on the calculation of partial coherences. The coherence between two time series indicates which frequencies (rhythms) are present in both time series. The graphical interaction modelling allows one to model feedback loops; both the direct and indirect relationships between therapy-relevant variables can be identified. In the literature, we find a few applications of this method to the analysis of electronic diary or monitoring data. Gather *et al*. [46] applied this method to the haemodynamic system of patients monitored in intensive care; Feiler *et al*. [11] analysed the therapy process of fibromyalgia patients using such interaction graphs. However, the main difference of this method to the graphical VAR modelling approach is that the calculations are made in the frequency domain. Therefore, it can not differentiate between same-time and lagged relationships. In comparison, the graphical VAR approach leads to time-related results. These results are probably of more interest as well as better interpretable and illustrative because the dynamic relationships between the variables are calculated in the time range and not in the frequency range. That is, we do not only obtain information about the strengths of associations in a system of multivariate time series, we obtain also information about the direction of influence in a relationship. Using, for instance, this analysis method in the electronic diary studies that investigated the association between cortisol and pain symptoms or psychosocial variables, the mutual influences of these variables could be modelled, thus overcoming the limitation that only associations but not influence directions between variables could be determined [9,14].

Both methods - the graphical VAR and the graphical interaction approach - use interaction graphs to elucidate the dependence structure in a system of variables. The mutual dependencies and directed influences in a system of variables may be complex and not easy to interpret. The visualization of temporal relationships among several variables in a clearly directed graph fosters an intuitive understanding of a complex dependence structure in a system of variables. Illustrating the model structure in a graph may help to clarify structural differences between various models and identify equivalences among them [23,46].

In our application study, data was collected only once per day. However, many EMA studies collect multiple measures per day. The fitting of a VAR model depends on the sampling frequency. For example, if a variable responds to changes in another variable very fast compared to the sampling frequency such an interrelationship will not show up in the autoregressive structure but only in the contemporaneous correlation structure. Thus, in principle, a high sampling frequency would be desirable. However, more frequent sampling also requires higher motivation of the participants. It would probably not be feasible to sample over several month with a high frequency per day. In general, some extra care should be taken when adapting the proposed approach to data from EMA studies with multiple measurements per day. There are two potential problems: First, measurement points are often not equally distant, and, second, circadian rhythms may be present. If measurement points during daytime are approximately equally distant and frequent enough, one could view the data as very short time series (longitudinal data) with different days being treated as (approximately) independent repetitions per subject. This would solve the absence of measurements during nighttime. Additionally, circadian rhythms could be modeled by a time-dependent deterministic mean. More generally, the interrelations among the variables may themselves be affected by circadian rhythms and thus be modeled as time-varying. Such general models, however, become soon computationally as well as--because of the large number of parameters--statistically infeasible for typical EMA studies. Therefore, in an electronic diary study, advantages and disadvantages of different sampling frequencies regarding the research question and analyses methods must be weighed against each other. In the following we want to discuss several possible limitations of the proposed method and application study.

Firstly, an essential feature of the graphical VAR method is that it makes Granger causality operational. As a limitation, we have to emphasize that Granger causality is not true causality. It merely states that prediction is improved if the entire information at the previous time point is included in the model. This definition inherently provides the limitation of all real-world studies - probably, we are not able to measure the entire information at the previous time point. That is, the VAR modelling is open to confounding from unmeasured variables. Nevertheless, knowledge about temporal--if not causal--influences could lead to the conceptualisation of new treatments to target specific variables which temporally precede changes in symptoms. If confounding is to be taken into account, a different modelling approach has to be used. One possibility would be Hidden Markov models, which explicitly include latent confounding variables in the model. However, as we do not know which associations among the variables might be affected by confounding, this would lead to an enormous amount of possible models thus increasing the computational burden of the model selection task by a multiple. Another possibility for identifying the causal structure of a system possibly affected by confounding has been proposed by one the authors [47]. However, efficient algorithms for implementing this approach still need to be developed.

Secondly, a limitation of the graphical VAR method could be seen in the selection approach. At the end of the exhaustive search over all possible constrained VAR models, one "optimal" model is selected although there could be many models that describe the data almost equally well. However, this uncertainty can be evaluated by significance tests; the discrimination between stable and unstable edges gives an additional basis of decision-making for the selection of the best fitting model.

A further limitation could be seen in that we estimated structural coefficients for a whole patient group. However, subjects probably differ in their structural coefficients, even within the same group. There are two possibilities to model such subject-specific differences in the structural parameters. First, a common structural VAR model can be fitted to each subject individually by imposing the same zero constraints on the coefficients (thus defining the structure) while estimating the unconstrained coefficients for each patient separately. The implementation of this generalization would be straightforward but has the disadvantage that due to the increased number of parameters more measurements per subject are required. In our application study we did not have enough measurement points to estimate such a large number of additional parameters. The second possibility would be a random effects VAR model where the subject-specific structural coefficients are treated as random with a common mean which then would be the parameter of interest. Fitting of such models requires more advanced and computationally more demanding estimation algorithms.

Lastly, as mentioned above, our approach focuses on the linear relationships among the variables. This includes the correlation as a measure for contemporaneous associations. Modeling conditional independences in multivariate responses is an open problem. In absence of a more general model, the VAR approach is a feasible alternative which yields reasonable approximations.

For medical research, one of the main advantages of the application of the graphical VAR method is the possibility to model feedback mechanisms. In many contexts, we have to assume that feedback loops in variable systems exist. Process patterns and temporal dependencies can be differentiated for specific subgroups of patients which could lead to differentiation and improvement of treatments. The present application study gives a good example of the new possibilities to characterize patient groups during treatment. The findings of the application study in obese patients with and without BED indicate that the two subgroups are distinguishable by the patterns which influence their respective eating behaviours. The findings support previous claims that the treatment of patients with BED should be focused not only on the improvement of disordered eating behaviour, but also on the improvement of depressive symptoms [48,49]. The application results point out that the use of the graphical VAR approach for the analysis of electronic diary data leads to a deeper insight into patients' dynamics and dependence structures. An increasing use of this modelling approach could lead to a better understanding of complex psychological and physiological mechanisms in different areas of medical care and research.

## Competing interests

The authors declare that they have no competing interests.

## Authors' contributions

The modelling and data analysis has been performed by BW and ME. HCF, SZ, MH and WH were responsible for the clinical conduct of the BED study and made contributions to the development of the manuscript. All authors read and approved the final manuscript.

## Pre-publication history

The pre-publication history for this paper can be accessed here:

http://www.biomedcentral.com/1471-2288/10/28/prepub
